# Clinical experiences of guided tapering of antipsychotics for patients with schizophrenia– a case series

**DOI:** 10.1186/s12888-024-05699-y

**Published:** 2024-03-29

**Authors:** Sofie Norlin Mølgaard, Mette Ødegaard Nielsen, Kickan Roed, Jimmi Nielsen

**Affiliations:** 1 Mental Health Center Glostrup, Copenhagen University Hospital, Unit for complicated schizophrenia, Glostrup, Denmark; 2 Mental Health Centre Glostrup, Copenhagen University Hospital– Mental Health Services CPH, Centre for Applied Research in Mental Health Care, Glostrup, Denmark

**Keywords:** Schizophrenia, Antipsychotic medication, Dose reduction, Tapering, Recovery, Relapse

## Abstract

**Background:**

80% of patients value information on treatment options as an important part of recovery, further patients with a history of psychotic episodes feel excluded from decision making about their antipsychotic treatment, and on top of that, mental health staff is prone to be reluctant to support shared decision making and medication tapering for patients with schizophrenia. This case series aims to demonstrate the tapering of antipsychotic medication and how guided tapering affects the patient’s feeling of autonomy and psychiatric rehabilitation.

**Case presentation:**

We present six patients diagnosed with schizophrenia (International Classification of Mental and Behavioral Disorders– 10th Edition codes F20.0–5, F20.7–9) who underwent professionally guided tapering in our clinic. The clinic aims to guide the patients to identify the lowest possible dose of antipsychotic medication in a safe setting to minimise the risk of severe relapse. Two patients completely discontinued their antipsychotic medication, two suffered a relapse during tapering, one chose to stop the tapering at a low dose, and one patient with treatment resistant schizophrenia, which is still tapering down.

**Conclusions:**

Reducing the antipsychotic dose increased emotional awareness in some patients (*n* = 4) helping them to develop better strategies to handle stress and increased feelings of recovery. Patients felt a greater sense of autonomy and empowerment during the tapering process, even when discontinuation was not possible. Increased awareness in patients and early intervention during relapse may prevent severe relapse.

**Impact and implications:**

Some patients with schizophrenia might be over medicated, leading to unwanted side effects and the wish to reduce their medication. The patients in our study illustrate how guided tapering of antipsychotic medication done jointly with the patient can lead to improved emotional awareness and the development of effective symptom management strategies. This may in turn lead to a greater sense of empowerment and identity and give life more meaning, supporting the experience of personal recovery.

## Background

Antipsychotic medication remains a cornerstone in the management of psychotic symptoms [1, 2]. With one in three patients possibly experiencing persistent symptoms despite antipsychotic treatment [3], the beneficial effect of long-term antipsychotic maintenance treatment for people with schizophrenia has been questioned [4, 5]. Qualitative research shows that people with schizophrenia often have the desire to reduce or discontinue their antipsychotic medication [6, 7]. Medication non-adherence among patients with schizophrenia is 41–49%, [8] and 47% of patients suffering from first-episode-schizophrenia will discontinue their antipsychotic medication within the first year of medication [9] despite sudden discontinuation possibly leading to exacerbation of symptoms, relapse, rehospitalisation, functional decline, and increased risk of death [10].

During recent years, an increased number of studies have focused on antipsychotic dose reduction [11–15] and deprescribing guidelines have also been published [16, 17].

This has led to a slight shift in recommendations and clinical guidelines towards dose reduction or possibly discontinuation [18], however, discontinuation is still increasing the risk of clinical worsening significantly [19].

Although research shows that 80% of patients believe that having information about treatment options is a crucial aspect of recovery [20], individuals with a history of psychotic episodes often feel excluded from decision making about their antipsychotic treatment [7]. Moreover, mental health staff may be reluctant to support shared decision making and the tapering of medication for patients with schizophrenia [21]. Shared decision making, which is defined as two people, in this case a psychiatrist and a patient, sharing information to build consensus about the preferred treatment before agreeing on a treatment plan, [22] has been associated with the alleviation of symptoms, improved self-esteem, increased satisfaction with medical care, better treatment adherence, and lower rates of hospitalisation [23].

Currently, randomised controlled trials investigating the effect of antipsychotic medications largely focus on relapse prevention [24] but fail to incorporate outcomes that reflect personal recovery [25], which according to the CHIME framework comprises the five components its name stands for: connection to peers, hope, identity, meaning of life, and empowerment [26]. To our knowledge, there are no studies that describe how guided tapering unfolds in clinical practice. The case series presents how structured and closely monitored tapering can affect various life domains and how providers of psychiatric rehabilitation can support the decision to reduce medication with the least risk of relapse. Our discussion of the potential benefits and difficulties of tapering reflects that the patients in our study were diverse and had different outcomes.

Keywords: schizophrenia, antipsychotic medication, dose reduction, tapering, recovery, relapse.

## Methods

In 2018, the health authorities in Denmark and the Capital Region of Denmark supported the establishment of a specialised outpatient clinic to meet the wishes of patients who wanted to taper their medication. The clinic offers guided tapering of antipsychotic medication for patients 18–64 years of age diagnosed with schizophrenia using International Classification of Mental and Behavioral Disorders– 10th Edition (ICD-10) codes F20.0–5, F20.7–9 [27] and works with patients to identify the lowest suitable dose of medication in a safe and professionally guided setting to minimise the risk of severe relapse.

Patients can be enrolled regardless of initial symptom load and level of function but to ensure the safe tapering of medication, patients must adhere to their medication and individual tapering schedule, as well as be available to speak by telephone weekly and to attend monthly appointments at the clinic. Further, we have a few exclusion criteria: Psychiatric hospitalization during the last 6 months, substance abuse to a degree which affect the possibility of participating in the regular assessments, and acutely increased risk of suicide or violence evaluated. In addition, the patient may be excluded if, following a comprehensive interview, the psychiatrist conclude that tapering is not recommended due to safety issue.

During the monthly visits, experienced and trained healthcare professionals evaluate symptoms based on the semi-structured interview using; the Positive and Negative Syndrome Scale (PANSS) [28]. We aim to reduce the antipsychotic dose monthly by 10%, and once half of the initial dose is reached, the dose is reduced monthly by approximately 5% until further reduction is no longer possible. Under the guidance of the staff, the patient decides whether to reduce the dose or not based on a thorough evaluation of their symptoms. When the patient decides to stop the tapering, or reach discontinuation, the patient is observed for additional 6 months. Although various models are available to ensure shared decision making [29], we do not adhere to any specific shared decision-making model.

Our unit’s staff strives to listen and comprehend the perspective and life circumstances of patients, while also connecting their experiences to past events and incorporating insights from other patients and scientific research. We are currently collecting data for a qualitative study on how our patients experience our approach during the tapering process.

The prescribing clinician collected data and case quotations at the monthly visits. The cases were meticulously selected to represent a broad range of tapering.

## Patient presentation

### Patient A

Patient A is a 29-year-old male diagnosed with paranoid schizophrenia (ICD–10 code F20.0) at age 20 who has experienced anxiety and tics in stressful situations since age 10. His positive symptoms comprised vague evil thoughts that he did not see as his own, inner dialogue interpreted as internal hallucinations, self-referential thoughts, and the delusion of being persecuted. In addition to experiencing sleep disturbances and avolition, he was described with blunted affect. For several years he received long-acting injectable (LAI) aripiprazole and presented with no psychotic symptoms but continually had severe negative symptoms, e.g., a lack of energy, anhedonia, and avolition.

Patient A was enrolled in our clinic at his request because he had increasingly experienced a lack of energy and motivation over the past five years. At enrolment he had no psychotic symptoms but was severely affected by negative symptoms. He did not work or study but was living independently.

Patient A received LAI aripiprazole 400 mg/month and had a PANSS score of 70, dominated by negative symptoms with a score of 26. He followed the algorithm for dose reduction and did not experience any change in psychotic symptoms. After 10 months, he stated, “*I feel like I may have been stuck in a repetitive narrative of my symptoms. While answering your questions, it occurred to me that there are very few symptoms left.*” After 12 months the medication was discontinued after the last aripiprazole injection of 80 mg, and after an additional 6 months of observation, Patient A stated that his emotional life had returned since he could feel joy and sadness again (PANSS 38, *P* = 7, *N* = 11, G = 20).

During the observation period subsequent to discontinuing the medication, some of his tics reoccurred, e.g., clicking his tongue up to 20 times a minute, but he was not affected to a level that required treatment. He worked therapeutically to give his life more structure, especially by keeping a regular diurnal rhythm. At the final clinic visit, Patient A explained that he had been approved for an early retirement pension but that he volunteered at a computer repair shop twice a week and that his social life had expanded. He has currently been without medicine for 12 months and still does not have any psychotic symptoms.

### Patient B

Patient B is a 41-year-old man who was referred for tapering after > 10 years of treatment with aripiprazole. At the age of 28, he was diagnosed with depression and treated with citalopram 30 mg/day and aripiprazole 5 mg/day due to ruminating and racing thoughts. During the following two years, he suffered a severe loss of function, affective flattening, anhedonia, avolition, self-neglect, and social withdrawal, and only partial symptomatic improvement was observed. Patient B was enrolled in a specialised assertive early intervention program [30] with close contact for five years, during which time his diagnosis was changed from depression to unspecified schizophrenia (ICD–10 code F20.9) due to hypnogogic hallucinations.

The aripiprazole dose was increased to 30 mg/day and then switched to LAI aripiprazole 400 mg/month. Side effects continued, including increased sleep duration and a 13 kg weight gain. A general practitioner (GP) treated patient B for four years to help manage symptoms and reduced the dose to 300 mg/month, whereupon the patient was referred to further tapering at our clinic.

Patient B wanted to taper off medication because he did not feel sick and was in doubt about why he still received medication. In addition to feeling exceedingly tired and sleeping around 14 h a night, he found it impossible to lose weight while on medication. When enrolled in the tapering program, patient B’s PANSS total score was 66, with a negative symptom score of 20 and a general symptom score of 36. He followed the tapering plan, and his last aripiprazole injection of 80 mg was given 10 months after enrolment. His PANSS rating declined to 52 (*P* = 9, *N* = 20, G = 23), mainly due to changes in general symptoms and he now slept only 9–10 h/night. He experienced that, “*the bell jar around him was removed*” and that he suddenly “*had to deal with his emotional life again*”, which worsened his tendency to ruminate about discussions with friends when going to sleep. To specifically address this issue, he was taught strategies in a focused therapeutic relationship before being discharged from the clinic. Patient B has now been without antipsychotic medication for 14 months and has no psychotic symptoms but has type 2 diabetes and hypertension. He regularly works out at a fitness center but continues to struggle with how to manage his emotional life and ruminating before he goes to sleep.

### Patient C

Patient C is a 35-year-old woman who was hospitalised at the age of 22 with visual and external auditory hallucinations with mixed second- and third-person perspective. She was diagnosed with paranoid schizophrenia (ICD–10 code F20.0) and treated with quetiapine 1600 mg/day, which was later changed to aripiprazole 30 mg/day and quetiapine 100 mg/day. After being stable for three years on LAI aripiprazole 400 mg/month, she resumed her education but experienced increased symptoms in terms of thought broadcasting, anxiety, and social isolation and had to interrupt her studies. The symptoms improved after adding quetiapine 200 mg/day, and after being stable for a period, quetiapine was changed to a pro re nata prescription, and aripiprazole was switched to an oral prescription, allowing her to resume her studies.

Patient C entered our clinic on her own initiative after being stable on aripiprazole 20 mg/day for one year. She was still hearing what she considered a good and supportive voice for the last 13 years, and she wanted to find out how well she could do with little or no medicine. Her PANSS baseline score was 40, with a positive symptom score of 10 resulting from a score of four at P3 (hallucinations). In the first week of tapering she said she was more emotional but that diminished before the next dose reduction. After six months, she was on a dose of aripiprazole 5 mg/day, which she chose to continue with even though she was about to move and had to terminate her treatment at the clinic. This decision was made after thorough consideration and discussion with the staff. Patient C explained that she was now “*more in touch with her feelings and her surroundings*” and her boyfriend said that she was also “*more emotionally available*”. Patient C appreciated having close contact with the staff while reducing her dose because it made her feel safer during the process. At this point, Patient C’s dose of aripiprazole 5 mg/day has been stable for four years. She is married and gave birth two years ago without her condition deteriorating. Patient C asserts that the dose reduction made her much more self-confident and “*in charge of her own life*”. She describes how being more in touch with her feelings makes it possible for her to have her own opinion, to judge what is good or bad for her, and that this has made her contact with reality stronger. She still hears a supportive voice in her head but it does not disturb her, and she no longer drifts into a psychotic reality. Her GP suggested stopping her medication completely, but she does not dare to do this without working closely with mental healthcare staff based on how safe and secure she felt while tapering her medication at the clinic.

### Patient D

Patient D was enrolled in the clinic at age 26 because he wanted to discontinue his antipsychotic medication. He was 18 years old when diagnosed with paranoid schizophrenia (ICD–10 code F20.0), and his positive symptoms included auditory hallucinations of second- and third-person perspectives, thought broadcasting, and persecutory delusions. At the time of enrolment, he did not have any symptoms of psychosis and had a total PANSS score of 34. He worked 32 h a week as a peer support worker and lived with his girlfriend. Patient D wished to discontinue the antipsychotic treatment mainly because he believed he had learned to cope with the symptoms through his recovery gains.

At enrolment, Patient D received aripiprazole 10 mg/day, which was reduced to aripiprazole 7.5 mg/day (P aripiprazole < 300 nmol/L, ref: 300–1700 nmol/L) during the first month. Patient D revealed that he had not been taking his medication regularly. To improve adherence, he agreed to switch to LAI aripiprazole 200 mg/month, which was tapered down to 120 mg/month over the next four months. Patient D started experiencing auditory and visual hallucinations, racing thoughts, and depressive delusions about being a hypocrite, his PANSS total score increased from 34 to 40. We practiced watchful waiting, i.e., we did not increase the antipsychotic dose unless symptoms increased, while Patient D went on sick leave.

At his next appointment at the clinic, we suggested adjusting the dose to LAI aripiprazole 300 mg/month. Patient D agreed that this was necessary, even though he was disheartened that he was unable to discontinue the medication, “*I thought I could do without. I tell the patients every day at work that to achieve recovery they need to fill their lives with meaningful things, but this shows that I just need to take the medication, nothing else matters*”.

The symptoms improved with the increased dose. After eight weeks, he was working 32 h a week again and had a PANSS total score of 31, which was lower than when he was enrolled at the clinic. Patient D told us that he felt he was “*functioning on a better level*” than when he began tapering. When we asked about his opinion on tapering, he replied: “*I think the time in the clinic has been good. I’m sad that I couldn’t do without medication, but now I know medication is important for me*”. He still receives and feels stable on LAI aripiprazole 300 mg/month.

### Patient E

Patient E is a 59-year-old woman who was diagnosed with paranoid schizophrenia (ICD–10 code F20.0) at the age of 27, presenting with persecutory delusions, auditory and visual hallucinations, sadness, and anxiety. She was enrolled in the clinic because she wanted to discontinue her medication since she questioned her need for antipsychotic treatment and whether her diagnosis was correct.

At the time of enrolment, Patient E had a PANSS total score of 59, her suspicion was easily aroused, she had trouble planning, and experienced anxiety. She lived on her own, had an adult daughter, and was working 11 h a week in a supermarket.

Patient E described her previous experience with treatment in the mental health services, “*I was hospitalised in Spain on a vacation, and they just injected me with medication. I don’t know which one. I was so scared, and now I don’t remember anything.*”

Patient E received LAI aripiprazole 200 mg/5th week at enrolment. We changed the dose to 160 mg/month, and Patient E began psychotherapy. After two months, her PANSS total score had increased to 62, mainly due to a five-point increase in the positive symptoms score, where P6 (suspiciousness) had increased by three points. She described her brain as stressed; she felt like she was being watched and was afraid of other people. Because of the minor increase in symptoms, we arranged an additional visit after 14 days and refrained from further dose reduction.

At the additional visit, she had a PANSS total score of 56 and told us that everything had cleared up but a week later she called and said that she needed additional medication. Patient E felt destabilised by intrusive thoughts about the time she got sick, a sense of derealisation, increased persecutory delusions, and a feeling of somebody speaking to her. As a result, we added aripiprazole 5 mg/day. At the next visit to the clinic, the PANSS total score had increased to 67 and the aripiprazole dose was increased to 300 mg/month plus 5 mg/day. She was nonetheless hospitalised in an open ward for 11 days without further changes in the medication. One month later, she started working again, and two months later she was stable, with a PANSS score of 48.

She concluded: “*Now I know that I need the medication and that I have a mental disorder. The relapse was very difficult, but it’s over now*.” Even so, she asked for a further dose reduction the next month because she believed that the medication was causing pain in her legs. She still receives LAI aripiprazole and individual therapy with a psychodynamic focus to support her understanding of her symptoms related to her previous life experiences.

### Patient F

Patient F, a 30-year-old male diagnosed with paranoid schizophrenia (ICD–10 code F20.0) at the age of 20, was hospitalised after a sudden psychotic breakdown, after which he described delusions of self-reference and surveillance. Initially, he received an eight-week treatment of aripiprazole 20 mg/day, but due to lack of effect this was changed to risperidone 6 mg/day. The delusions disappeared gradually, and his positive symptom load was described as in remission after two years. Patient F experienced side effects such as weight gain (30 kg), increased sleep, apathy, akathisia, hyperprolactinemia, and sexual dysfunction. At his initiative, he discontinued the medication abruptly. Four months later, Patient F was hospitalised because he had covered his windows with oil, tomatoes, and flour to protect himself due to his delusions. Treatment with risperidone was resumed with some effect, but he refused treatment with long-acting antipsychotics. After a few months, Patient F discontinued the medical treatment and refused to have any contact with the mental health services. One year later, he was hospitalised after believing that he had clairvoyant and telepathic abilities. He initially received olanzapine 40 mg/day for two weeks, then zuclopenthixol 40 mg/day for six weeks, and after that, sertindole 24 mg/day for five months, all with insufficient effect. Subsequently, he received clozapine 600 mg/day (P-clozapine 2802 nmol/L, ref: 300-2000nmol/L) without any further improvement, which was augmented with amisulpride 400 mg/day. Since this did not change that he believed that he had clairvoyant abilities, he received 18 electroconvulsive therapy treatments, also without any effect. After being hospitalised for 20 months, Patient F was discharged to his apartment but with daily visits to ensure continuous medication adherence in the form of clozapine 425 mg/day and amisulpride 250 mg/day. During the following three years, he was stable, while clozapine was gradually reduced to 175 mg/day before he was referred for further dose reduction at our clinic.

Patient F wished to discontinue clozapine as he disagreed with the diagnosis and experienced side effects in terms of weight gain (body mass index of 33), newly diagnosed diabetes, and constantly feeling tired. He lived a quiet and socially isolated life, mainly filled with telepathic contacts, and he was still convinced that he had clairvoyant and telekinetic abilities. His initial PANSS total score was 85, with a positive symptom score of 27. After 16 months of enrolment at the clinic, his clozapine had been reduced to 12.5 mg/day without any increase in symptoms or decrease in level of function. Patient F has felt a slight improvement in that he experiences less emotional indifference, with a decrease in PANSS negative score from 26 to 22. Patient F still visits the clinic and aims to stop clozapine within the next four months, after which the plan is to follow him for six months on a stable dose of amisulpride 250 mg/day.

## Discussion and conclusions

The benefits of tapering antipsychotic medication are under debate [31], but many patients request it, despite critics’ contention that it puts patients at unnecessary risk of relapse [32, 33]. However, determining whether the current antipsychotic dose is still needed can only be ascertained by reducing the dose, and, as most side effects are dose dependent, treatment with the lowest effective dose is of crucial importance [34].

Our case series illustrates how diverse tapering, and its outcomes can be while also demonstrating how an increased feeling of autonomy during tapering may lead to an increased feeling of rehabilitation and recovery, despite the outcome. For example, Patient A and Patient B were able to discontinue antipsychotic medication completely with no worsening of positive symptoms but an improvement in negative symptoms and quality of life. Both patients have now been out of antipsychotic medication for more than a year with no signs of worsening, which is considered a favourable predictor [35, 36], but as schizophrenia is seen upon as a life long condition the risk of relapsing after 12 months is still present. Many patients wish to reduce their dose or quit their medications entirely due to side effects, but this was only partly the case for Patient A and Patient B. When their antipsychotic medication was reduced and finally stopped, both patients experienced significantly more energy, initiative, pleasure, and emotional fluctuation. Although their files described them as “dominated by negative symptoms”, they appeared to be somewhat secondary to the medical treatment. Discriminating between primary and secondary negative symptoms can be almost impossible, but dose reduction can help to identify the negative symptoms medication causes. Patient A and Patient B increased their level of function and had a greater feeling of personal recovery in terms of hope, identity, the meaning of life, and empowerment [26]. Another important observation regarding the cases, especially Patient B, is that the level of symptoms led to long-lasting treatment with antipsychotic medication. Patient B’s files indicated that his medical treatment was initiated due to rumination and provided no evidence that he had suffered from delusions, hallucinations, or severe thought disorders. When guidelines recommend continuous antipsychotic treatment for all patients with schizophrenia, it categorises all patients as being at high risk for a severe psychotic episode. We argue that Patient B illustrates that this may not always be the case, underlining the importance of examining the individual patient’s history and evaluating the pros and cons of continuous treatment in collaboration with the patient to reach consensus.

Patient C, who had a similar outcome, decided to stay on 5 mg of aripiprazole with no signs of worsening. Although Patient C did not discontinue the antipsychotic drug completely, she experienced the benefits of the dose reduction and appreciated the tapering. Her decision to stop tapering her medications was partly due to moving to another part of Denmark, which required her to terminate treatment at our clinic. Patient C’s GP had suggested that she stop her antipsychotic treatment multiple times over the past years, but Patient C was aware that discontinuing the final dosage might worsen her symptoms and affect her overall level of functioning. As a result, she preferred to work with specialists to minimise any risks, which emphasises the importance of providing a safe and professional environment for tapering and shows that she felt that professional guidance in a specialised clinic decreased her risk of relapse. Consequently, although patients with no symptoms and no recurring symptoms during tapering seem to be the most suitable group for tapering, many other patients may benefit from a tapering trial.

Both Patient D and Patient E experienced relapses, the appearance of symptoms indicating that antipsychotic treatment was required. For Patient D, this led to psychologically accepting his psychiatric condition and the need for antipsychotic treatment. For Patient E, the impact on her perception of the necessity of medication may not be permanent. We are currently exploring whether a therapeutic relationship with a psychodynamic focus may be helpful to Patient E’s rehabilitation and lead to an increased feeling of personal recovery.

Patient F involved treatment-resistant schizophrenia; the various treatment strategies tried resulted in increased side effects rather than reduced psychotic symptoms. Often treatment is initiated during a worsening of symptoms, but disease fluctuations make evaluating the response difficult. In accordance with our experiences with this patient group, a Finnish study showed that olanzapine, combined with clozapine treatment, could be tapered with no worsening in symptoms [37]. Gradual tapering of antipsychotic drugs is not only relevant for patients with no symptoms but should also be considered for patients with treatment-resistant schizophrenia, where antipsychotic medication may not be efficient. In addition, treatment with clozapine may be associated with several troublesome side effects [38]. Today, only few existing guidelines address this issue [39, 40].

Several patents in our clinic describe increased emotional awareness or emotional fluctuation during dose reduction. Although we did not monitor the emotional awareness using a rating scale, this is in line with recent findings [41]. This restoration of emotional reactivity during dose reduction can be a temporary state patients need to go through, but it can also be a warning sign of relapse. Frequent clinical assessment, psychoeducation and therapeutic support may be needed, and it is our experience that sometimes the patients want to resume antipsychotic treatment or increase the dose, even though no psychotic symptoms have occurred. Although tapering is associated with an increased risk of relapse [32], this case series shows that intensive follow-up can be used to identify relapse at an early stage before it becomes more severe and devastating. Before inclusion in the tapering program, we thoroughly inform patients that tapering is associated with an increased risk of relapse, which is a concern that most patients share with us. However, the desire to reduce their medications exceeds the fear of relapse and, through common agreement, we ensure a therapeutic alliance where patients recount being heard and understood.

Our tapering program is one example of how to reduce medication with the least risk of relapse. Our findings show that taking advantage of specialised mental health services with close contact during gradual tapering may reduce the risk of severe relapse, at least compared to patients who independently initiated abrupt discontinuation. While we have not encountered suicide attempts, violent actions, or other serious complications so far, it is crucial to remember that tapering down antipsychotics carries a risk and must be approached with the utmost precaution.

Regardless of the outcome of tapering, patients regularly visiting the clinic were often grateful for the opportunity to have their antipsychotic medication reduced and to be involved in decisions about their medical treatment, which created a greater feeling of autonomy. We are in the process of collecting data for a qualitative study on how our patients experience our approach during the tapering process. Likewise, well-designed future qualitative studies are required, to shed further light on the benefits of using specialised mental health services with close contact during tapering.

This case series has various limitations, but one in particular must be acknowledged. The cases presented were deliberately chosen from a pool of more than 100 patients from two tapering trials. The presented cases were chosen to achieve a diverse sample and to illustrate some of the various ways in which tapering can occur but are not necessarily quantitatively representative. (The full data and results will be published in the following years) Consequently, the results are not generalisable to all patients suffering from schizophrenia.

Research on antipsychotic tapering remains sparse, and additional quantitative and qualitative data are warranted. Some of the patients in our cases who reduced their antipsychotic dose increased their emotional awareness and seemed to develop better strategies for handling stress, which may have led to an increased feeling of empowerment, identity, and meaning in life. Future research may resolve whether this feeling of personal recovery is a factor that improves tapering outcomes. Notably, some of the patients in our clinic were increasingly open to antipsychotic treatment during stressful periods, indicating that treating schizophrenia more in terms of episodes is warranted.

The diagnoses of schizophrenia cover a heterogeneous group of patients, making it crucial to take an individualised approach to meet the specific treatment requirements of each patient. Also, antipsychotic associated benefits and sideeffects should be carefully and regularly evaluated with the patient. If guided tapering of antipsychotics is considered, the possible risk of relapse and consequences of this should also be discussed.

## Data Availability

No datasets were generated or analysed during the current study.

## Abbreviations

ICD-10: International Classification of Mental and Behavioral Disorders– 10th Edition
PANSS: The Positive and Negative Syndrome Scale
LAI: Long-acting injectable
GP: General practitioner
